# Has the Cerebellum Any Special Connection with the Sexual Propensity of Function of Generation

**Published:** 1851-01

**Authors:** N. S. Davis

**Affiliations:** Member of American Medical Association; Prof. of Physiology and Pathology in Rush Medical College, Chicago, Illinois


### Part 3.—Selections.

### ARTICLE I.

*Has the Cerebellum any Special Connection with the Sexual Propensity or Function of Generation?* By N. S. DAVIS, M. D., Member of American Medical Association; Prof. of Physiology and Pathology in Rush Medical College, Chicago, Illinois.

Perhaps there is no part of the nervous tissue concerning the functions of which a greater variety of opinions are entertained than in relation to those of the cerebellum. Thus, Gall, Spurzheim, and the whole of that school of physiologists, represent it as one of the best settled facts in *phrenological* science, that the cerebellum constitutes the nervous centre, which presides over the sexual instinct, or functions of generation; while on the other hand, the vivisections of Flourens, Magendie, Bouillaud, &c., have led far the greater number of physiologists to regard this division of the encephalon as a special organ, for co-ordinating muscular movements. M. Foville, again, regards it as "the organ for the *perception* of muscular *sensibility*." The tables of M. Serres, and the investigations of Muller, Carpenter, and others, show, that comparative anatomy, on the whole, favors the experimental results of MM. Flourens and Bouillaud. Yet the well-known distrust with which we regard conclusions drawn exclusively from vivisectional experiments, or extensive injuries of the various parts of the nervous system, makes it desirable to avail ourselves of every other practicable mode of investigation. It is in this view that the importance of a careful study of developemntal and comparative anatomy cannot be too highly estimated.

Investigations based on well-known physiological laws are always valuable, even though their results may be entirely of a negative character. The law that appropriate exercise increases the growth of any organ or tissue, and that want of such exercise retards or entirely arrests such growth, is of universal application.

And by no class of men is this law more strenuously insisted on than by the Phrenological school, as applied to different parts of the brain. Hence we find Gall, and his followers, contending that injuries of the cerebellum not unfrequently cause the testicles to become atrophied, and *vice versâ.* Acting on the idea suggested by these *alleged* facts, M. Lassaigne, a few years since, made a comparison between the weight of the cerebellum in the stallion, gelding, and mare; all the animals examined being over ten years of age. The result, as is now well known, was that the cerebellum of the gelding was found heavier, both *absolutely* and relatively to the cerebrum, than that of the stallion and mare.

But the comparison of M. Lassaigne extended no farther than to the absolute and relative size of the whole cerebellum in mass. Hence, many phrenologists avoided the force of his conclusions by admitting that a *part only* of the cerebellum was connected with the instinct of generation or sexual appetite. And even Carpenter, in the very recent American edition of his "Principles of Human Physiolgy," holds the following language; "The author is far from denying, *in toto*, that any peculiar connection exists between the cerebellum and the genital system; but if the evidence at present adduced in support of phrenological position be held sufficient to establish it, in defiance to so many opposing considerations, we must bid adieu to all safe reasoning in physiology. The weight of testimony appears to him to be quite decided in regard to the connection of the cerebellum with the regulation of the motor function. *How far this invalidates the moderate phrenological view, which does not regard the function of the cerebellum as exclusively devoted to the sexual instinct, is a question well deserving attention.*" And on the same page he adds: "Further, it would seem by no means *improbable* that the *lobes* are specially connected with the regulation and co-ordination of movements; whilst the *vermiform processes*, which are very large in many animals, in which the former scarcely present themselves, are the parts connected with the sexual functions." (See *Carpenter*, p. 350, 4th edit.) Wishing to test the truth or falsity of this "*moderate phrenological view*," I recently procured the brains of several bulls and oxen, all of the same age, and as nearly as possible of the same general size and variety of stock. The cerebellum of each was carefully and accurately measured in the following directions, viz: First, *transversely*, from the lateral extreme of one lobe to that of the other. Second, *antero-posteriorly*, from the most anterior superior point of the central portion, to the most posterior inferior point lying over the commencement of the spinal cord.—Third, *vertically*, from the tip of the inferior vermiform process, resting on the medulla oblongata, to the most prominent point on the superior surface of the cerebellum. Fourth, from the apex of the inferior vermiform process to the apex or most prominent point of the superior veimiform process. Fifth, *transversely* across the widest part of the superior vermiform process. As the reader will readily perceive, it was my object to measure, not only the whole cerebellum, but also its several parts ; and especially its central or vermiform processes in comparison with its lobes. As the measurments uniformly tended towards one result, I shall occupy time and space to give in detail only the *average* of the measurments in each direction, and the extremes of the same. All the bulls and oxen examined were six years old ; though the bulls were actually heavier than any of the oxen whose brains were examined. The following is the result in tabular form, viz:—

| | Greatest Transverse Diameter | Greasest Antero posteri'r Diameter | Vertical Diameter | Length of Vermiform Processes | Breadth of Superior Vermiform Processes |
|---|---|---|---|---|---|
| Av'ge of Bulls examined | 32½ lines | 27¼ lines | 14½ lines | 16½ lines | 13¼ lines |
| Extremes, | 33 & 32 lines | 28½ & 26 lines | 15 & 14 lines | 18 & 15 lines | 13½ & 13 lines |
| Av'ge of Oxen examined | 32 lines | 27¾ lines | 16 lines | 19½ lines | 14½ lines |
| Extremes | 33 & 30 lines | 27 & 28½ lines | 16½ & 15 lines | 20 & 18 lines | 15 & 14 lines |

Average weight of the cerebellum of the bulls 13⅓ drachms. Average weight of the cerebum of the same, 115½ drachms. Proportion 1 to 8 3-5.

Average weight of cerebellum of oxen 13½ drachms.—Average weight of the cerebrum of same, 114 drachms. Proportion 1 to 8 1-5.*

It will be seen that these examinations not only confirm those of M. Lassaigne, so far as the relative and absolute weight of the cerebellum of the castrated animal is compared with that of the same age not castrated, but the measurments equally refute the *moderate phrenological view* mentioned by Carpenter, which, as we have already said, makes the central part and vermiform processes the organ of sexual appetite, and the lateral lobes the co-ordinators of motion. On the

* It should be stated that a much larger nnmber of brains of oxen are included in he foregoing examination than of bulls. Hence, while the average weight of all theirtcerebums appears, as stated, slightly less than those of the bulls, yet the heaviest cererum of the ox examined was fully equal to the heaviest of the bull.

contrary, so far as our measurments indicate any difference, it consists in the greater relative development of the central part, and vermiform processess in the ox than in the bull. And, if the cerebellum of the ox is deficient in any part, it is in the size of the *lateral lobes*. But, on the most careful examination of half a dozen cerebelli of bulls and oxen lying side by side, I could detect no constant or uniform variation either in their absolute size, or the relative development of their several parts. Now, unless we deny the validity of the physiological law, so much insisted on especially by phrenologists, that exercise increases growth, and *vice versa*, it must be conceded that the facts here adduced in reference to the cerebelli of oxen and bulls, added to those presented by M. Lassaigne in reference to the gelding and stallion, effectually disprove the existence of any *special* connection between the cerebellum, either, as a whole or in any of its parts, and the sexual appetite or function of generation. And, if we search the facts of comparative anatomy ever so rigorously, we shall find nothing to militate against the results of these investigations, but very much to confirm them. Hence, whatever of truth there may be in the phrenological location of other cerebral *organs*, we cannot credit even the *moderate views* of that school in regard to the functions of the cerebellum. It is much to be regretted that our most approved works on physiology still contain so many mere statements of opinions, the truth of which a little patient and careful investigation would directly demonstrate or disprove.—*Transactions of American Medical Association for* 1850.

